# St. Louis enhancing engagement and retention (STEER) in HIV/AIDS care: a participatory intersectional needs assessment for intervention and implementation planning

**DOI:** 10.3389/fpubh.2025.1589671

**Published:** 2025-06-11

**Authors:** Debbie L. Humphries, Phillip Marotta, Yue Hu, Victor Wang, Greg Gross, Darius Rucker, Johnnie Jones, Faiad Alam, Tawnya Brown, Donna Spiegelman, Chelsey R. Carter

**Affiliations:** ^1^Department of Chronic Disease Epidemiology, Center for Methods in Implementation and Prevention Science, Yale School of Public Health, New Haven, CT, United States; ^2^Brown School, Washington University in St. Louis, St. Louis, MO, United States; ^3^Yale School of Public Health, New Haven, CT, United States; ^4^Keys to Knowledge and Action Consulting, St. Louis, MO, United States; ^5^St. Louis Ryan White Planning Council, St. Louis, MO, United States; ^6^Vivent Health, St. Louis, MO, United States; ^7^Department of Biostatistics, Center for Methods in Implementation and Prevention Science, Yale School of Public Health, New Haven, CT, United States; ^8^Department of Social and Behavioral Sciences, Yale School of Public Health, New Haven, CT, United States

**Keywords:** participatory planning, intersectional needs assessment, intersectionality, implementation planning, ending the HIV epidemic

## Abstract

**Background:**

Missouri is one of seven priority states identified by the Ending the HIV Epidemic Initiative, and St. Louis contains almost half of the people living with HIV (PLWH) in Missouri. As St. Louis has a marked history of structural racism and economic inequities, we utilized the Intersectionality Based Policy Analysis (IBPA) framework to guide a participatory needs assessment for planning and program development.

**Methods:**

The planning team included researchers, the lead implementer from our community partner, and two community representatives, and had biweekly 60–90 min meetings for 18 months. The planning team approved all research materials, reviewed and interpreted results, and made decisions about recruitment, conduct of the needs assessment, and development of the planned intervention. The needs assessment integrated information from existing data, (1) interviews with (a) PLWH (*n* = 12), (b) community leaders (*n* = 5), (c) clinical leaders (*n* = 4), and (d) community health workers (CHWs) (*n* = 3) and (e) CHW supervisors (*n* = 3) who participated in a Boston University-led project on CHWs in the context of HIV and (2) focus groups (2 FG, 12 participants) with front-line health workers such as peer specialists, health coaches and outreach workers. A rapid qualitative analysis approach was used for all interviews and focus groups.

**Results:**

The IBPA was used to guide team discussions of team values, definition and framing of the problem, questions and topics in the key informant interviews, development of the logic model of the problem, and all results. Applying the IBPA framework contributed to a focus on intersectional drivers of inequities in HIV. The effective management of HIV faces significant challenges from high provider turnover, insufficient integration of CHWs into care teams, and organizational limitations in tailoring treatment plans. Increasing use of CHWs for HIV treatment and prevention also faces challenges. People living with HIV (PLWH) encounter multiple barriers including stigma, lack of social support, co-morbidities, and difficulties in meeting basic needs.

**Conclusion:**

Addressing intersectional drivers of health inequities may require multi-level, structural approaches. We see the IBPA as a valuable tool for participatory planning that emphasizes equity and integrates community engagement principles in program and implementation design for improving HIV outcomes.

## Highlights

- We report an innovative process for conducting a participatory intersectional needs assessment as an initial phase of intervention mapping and implementation mapping.
- The intersectionality-based policy analysis framework provided a structured approach to identify structural and systemic barriers to the effective management of HIV.
- Barriers to effective management of HIV were identified at multiple levels and included high staff turnover, insufficient numbers of CHWs, and insufficient integration of CHWs at the clinic and institutional levels.
- The participatory team developed two complementary logic models of the problem to incorporate both systemic drivers of inequities and the multiple pathways where positionality and intersectional barriers impact HIV care outcomes.

## Background

Ending the HIV epidemic is a critical priority for global public health (1–3). Despite progress in the prevention and treatment of HIV over the past decades, new infections occur daily in the United States and are highest among Black men, particularly those who have sex with men (4). A significant number of people living with HIV (PLWH) in the United States are not receiving adequate care, with an estimated 21% being unaware of their HIV status, 25% not linked to care within 6–12 months of diagnosis, 50% not engaged in routine care/follow-up, and 25% not receiving antiretroviral therapy (ART) (4). Inequities in access to HIV treatment persist among young adults, people of color, and the lesbian, gay, bisexual, transgender, and queer (LGBTQ) communities, highlighting the importance of an intersectional lens in developing and improving interventions to mitigate the complex web of inequities (5–7).

To tackle these challenges, the Ending the HIV Epidemic Initiative (EHE) was launched in 2019 with the goal of a 90% reduction in new HIV infections in the United States by 2030 (1). The EHE prioritizes early diagnosis, rapid and effective treatment, prevention strategies such as pre-exposure prophylaxis, and quick response to potential outbreaks to make HIV a rare infection. Missouri is one of seven priority states under the EHE initiative due to higher rates of rural infections and presents unique challenges with a history of socioeconomic and racial inequities. In St. Louis Black men and women are three to eight times more likely to be diagnosed with HIV than their white counterparts (8–11), and high rates of HIV infection and lower engagement along the continuum of care are seen among young adults, people of color, and LGBTQ communities (5–7). There are notable gaps in understanding the complex and overlapping dynamics that influence engagement and retention in HIV/AIDS care. The lack of an intersectional lens has obscured the structural vulnerabilities created by systemic responses to race, gender, sexual identities, and socioeconomic status, hindering comprehensive solutions for diverse communities (9–11). Applying an intersectional lens to understand social and structural determinants of health through a participatory community approach can inform the adaptation and implementation of targeted interventions, such as Community Health Workers (CHWs) to address the complex and interconnected factors contributing to HIV inequities.

### Use of community health workers in HIV management

The deployment of CHWs to provide enhanced client support and strengthen client trust is an effective strategy for enhancing client engagement (12), providing acute treatment (13) and overcoming social and structural barriers that burden underserved populations (14). CHWs are trusted members of the local community who deliver critical health promotion interventions characterized by racial, socioeconomic, and ethnic backgrounds, as well as lived experiences that align with those of their client population (15–18). This strategy has improved health outcomes for chronic conditions such as diabetes (19) and has been a valuable tool in responses to the COVID-19 pandemic (20). In addition, CHWs have demonstrated a remarkable capacity to bridge gaps in the provision of HIV treatment as PLWHs working with trained CHWs have higher rates of engagement along the treatment cascade and better health outcomes (21–24). Enhanced personal contact between lay health workers and PLWH strengthens the continuity of care for HIV clients by developing closer bonds with healthcare providers and reducing distrust of the medical system (25). Integrating CHW roles into HIV treatment settings increases engagement in HIV treatment and care, although there are important challenges to their implementation, such as role ambiguity (26), the need for strong infrastructure support for CHW programs, including resources, recordkeeping and data-sharing systems (14, 27, 28), and career development opportunities for CHWs (29). More implementation research is needed to understand the best ways to implement and scale up the integration of CHWs into medical systems of care for PLWH (21, 30).

### Implementation science tools for planning

Intervention Mapping and Implementation Mapping (IntMap and ImpMap) (31) have been used extensively to strengthen intervention design and implementation planning. Together they provide a process for systematically developing an intervention and an implementation plan, beginning with a needs assessment to define the problem and develop a logic model of the problem (32–36).

### Participatory planning and needs assessment

Participatory approaches to intervention adaptation and implementation increase community buy-in and enhance the viability, effectiveness and sustainability of interventions (37–40). Participatory planning involves active participation and input from healthcare providers, community members, and other stakeholders to increase the likelihood that systems, service delivery, and implementation are tailored to the community, leading to better health outcomes and improved quality of life (41–45). Community participation in HIV research has a long history, formalized in the 1983 Denver Principles which called for formal and equitable engagement of community members often through community organizations and advisory boards (46–48). Participatory planning is critical for offering patient-centered HIV care that is responsive to the unique needs and preferences of the community (49, 50). By including PLWH in the process of adapting programs and implementation planning, teams can formulate more effective implementation strategies to strengthen the ability of CHWs and their organizations to reduce stigma and discrimination, increase treatment adherence, and enhance the ownership by PLWH for their care (49, 51). Participatory planning can help identify and address barriers and facilitators to care, such as lack of access to transportation, concerns about confidentiality, and levels of client-provider trust (41). The use of participatory approaches not only acknowledges the unique insights and experiences of those directly affected by HIV but also cultivates a sense of investment and collaboration within communities. By integrating an intersectional approach with participatory planning in conducting the needs assessment, we enhanced our ability to capture and respond to the community’s needs.

### Intersectionality-based policy analysis conceptual framework

**Figure 1:**
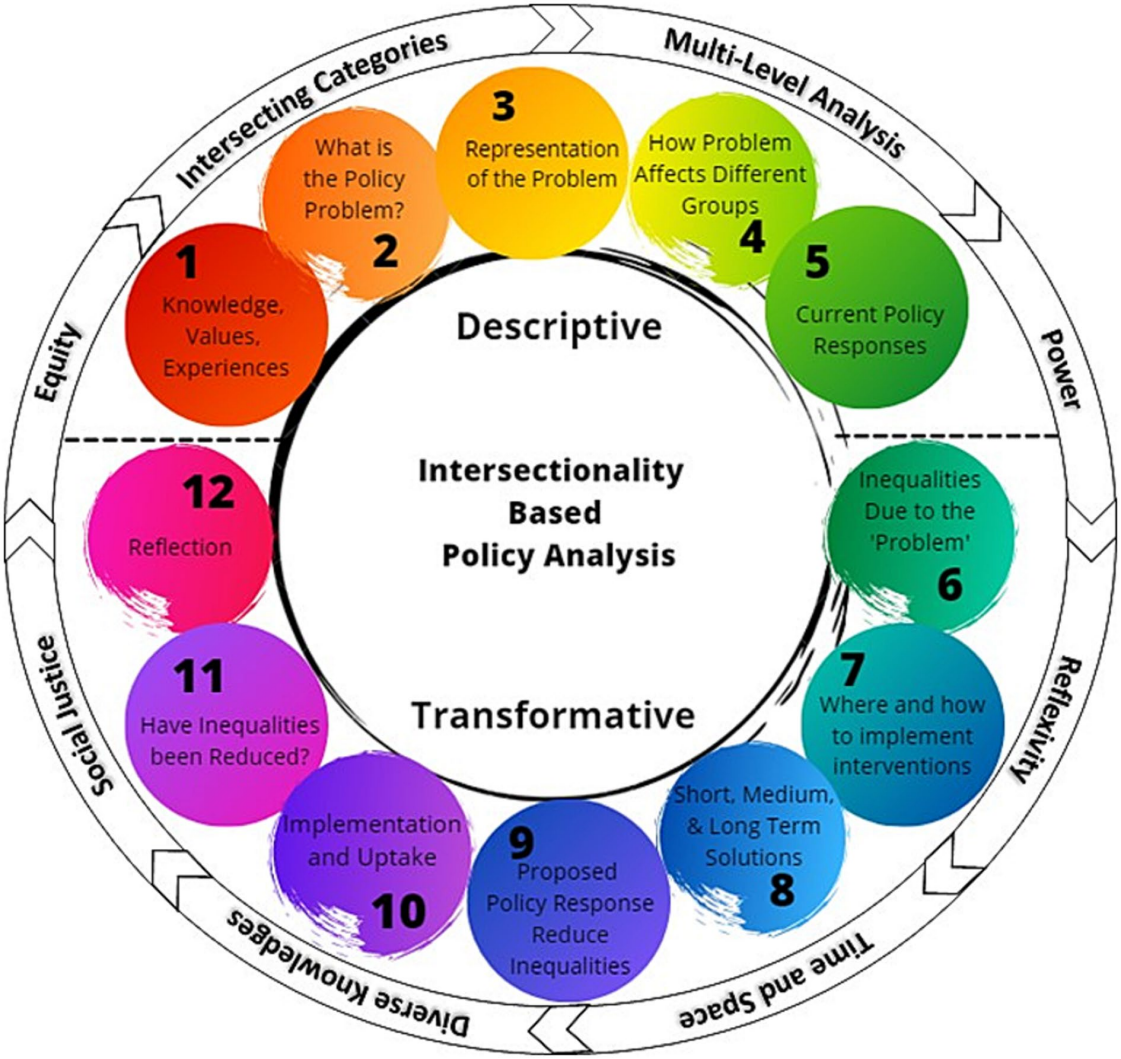
Intersectionality-based policy analysis framework. The descriptive and transformative questions are shown surrounded by the guiding principles to highlight the complex interaction of the two key components of the framework, and the importance of addressing both questions and guiding principles simultaneously [Intersectionality-Based Policy Analysis Framework© 2021 by Debbie Humphries, Michelle Sodipo, Skyler Jackson; based on ideas of Olivia Hankivsky is licensed under Attribution 4.0 International (60)].

We used the intersectionality-based policy analysis (IBPA) framework (52) to adapt a multilevel intervention and develop implementation strategies for a CHW-centered program to reduce inequities along the HIV care cascade. Intersectionality theory focuses on moving beyond examining individual factors, such as biology, socioeconomic status, sex, gender, and race, and elucidates the relationships and interactions among multiple interlocking systems across all levels of society for an individual or group (53–55). The IBPA framework (Figure 1) explicitly employs principles of social justice, power, and diversity of knowledge to interrogate overlapping systems and structures that affect policy and programmatic issues (56). IBPA has been applied in various case studies and settings, including maternity care, HIV prevention strategies for gay men, and the criminalization of HIV nondisclosure in Canada, generating equity-focused perspectives that incorporate diverse viewpoints in both defining problems and seeking sustainable solutions (52, 57–59). The IBPA framework has also been used to identify intersectional drivers of inequities in access to HIV treatment services (56). We selected this framework to guide intervention adaptation and implementation planning based on the explicit emphasis on discussing values and creating a participatory and equity-based space for leading the project, as well as an emphasis on praxis – using an intersectional lens to change systems (60–62). Recent studies have demonstrated the value of the IBPA framework (62–64), exemplified by its application to the United States COVID-19 policy response (60); which underscores the growing significance of considerations of intersectionality in public health and highlights the transformative shift from analysis to actionable strategies.

The St. Louis Enhancing Engagement and Retention in HIV/AIDS Care (STEER) project conducted a participatory intersectional needs assessment to adapt a CHW intervention to enhance the management of HIV/AIDS care and prioritize implementation strategies. We describe here a novel application of the IBPA within a participatory needs assessment that integrated community leaders into the research team to lay the groundwork for adapting and implementing a CHW-centered multi-level intervention to address the needs of PLWH in St. Louis.

## Methods

### Setting

The study took place in St. Louis, Missouri (9.2021 through 5.2023), which has a high burden of HIV among historically marginalized communities. St. Louis residents comprised almost half (6,320 of the 13,109) of persons living with HIV in Missouri in 2018. In 2020, the population of St. Louis (300,576 people) was 46.4% Black, 46.5% White, 4.0% Hispanic or Latino, 0.3% Native American, and 3.4% Asian (8). Approximately one in five people in St. Louis live below the poverty line.

### Research aims and design

The project utilized intervention mapping (IntMap) and implementation mapping (ImpMap) (31, 65) to adapt a CHW intervention and plan for its implementation. We report here on the initial phase, a participatory intersectional needs assessment utilizing the IBPA and development of a logic model of the problem of HIV care management and engagement. This report is intended to (1) articulate the internal participatory planning process using the IBPA guiding principles, (2) present the methods and results of primary data collection to examine barriers to engagement in HIV care, (3) present the multi-level conceptualization of the problem of HIV care management and engagement in St Louis and the potential for CHWs to address this problem using the IBPA framework prompts, and (4) present the culmination of these analyses as a set of two ‘Logic Models of the Problem’ as specified in the first phase of intervention mapping. The planning process and the results are intentionally presented together to demonstrate the organic process utilized for constructing research results, as posited by intersectionality theory (66).

### Research process

#### Building the research team

The research team developed organically and intentionally by connecting with potential partners in St. Louis through existing networks of trust. Two academic partners and a community-based organization (CBO) leader with decades of experience in the St. Louis HIV treatment and prevention community jointly agreed to investigate how best to strengthen the implementation of CHW services in the context of HIV treatment. The CBO leader identified two trusted and innovative leaders in the PLWH community, and both met first with one of the academic leads to discuss the research plan, including the IBPA framework and participatory planning approach, and to address any questions. Both chose to join the bi-weekly planning team meetings as consultants.

The nested team structure included a planning team consisting of CBO leadership, community leaders, and university-based faculty and research assistants who met bi-weekly to develop research materials, review progress, address ongoing needs, and guide decision-making. Planning team members were selected to bring both topical expertise and a range of lived experience to the project. The full research team, including experts in community health worker health program design, geographic modeling, and qualitative and quantitative research, were consulted to help synthesize insights from the research and plan the implementation. Local leaders on the St. Louis Fast Track Cities (FTC-STL) Steering Committee also provided a strong community perspective during the project. The FTC-STL brings together academic, government, CBO, and community leaders in the field of HIV in St. Louis to strengthen the St. Louis response to the HIV epidemic, working together to achieve the global goal of the Fast Track Cities movement, to end AIDS as a public health threat by 2030 (67). The team shared initial findings with the FTC-STL Steering Committee to gather feedback.

#### Applying the IBPA

There are two primary components of the IBPA: guiding principles and questions (52, 57). Eight guiding principles are provided to consider and integrate into the analytical process, along with 12 analysis questions. The first set of questions (Q1-5) are descriptive and focus on the team, the problem to be addressed, how the problem is framed, the impacts of the problem, and current policy responses. The subsequent questions (Q6-12) are transformative, addressing inequities in current impacts of the problem, potential solutions, ensuring implementation, metrics of success, and the team’s reflections on the use of the IBPA. Definitions of guiding principles were adapted from Humphries et al. (60) and the planning team identified examples of how each principle was applied by the planning team in conducting the needs assessment. Draft answers to the first five questions of the IBPA were developed within the first 6 months of the planning team process, and informed development of the interview guide and specific subgroups we sought to include in the interviews. Draft answers for all IBPA questions were developed by subgroups of the planning team and shared with the entire planning team for feedback before approval. Responses to the IBPA questions were developed in parallel with data collection and analysis, with the IBPA guiding data collection and analysis.

#### Planning team activities

Early in the timeline (9.2021–5.2023) the planning team devoted a session to each planning team member sharing their knowledge, values, and experiences that contributed to their interest in this project (IBPA Q1: *What knowledge, values, and experiences do you bring to the area of analysis?*). Results were synthesized, reviewed, and approved by the planning team. Other activities included reviewing timelines, draft materials (such as IBPA responses, interview guides, recruiting materials, and potential participants, analysis results, problem logic model), providing feedback on results, providing insights into community history and current community activities, as well as strategies to strengthen the implementation of CHWs in the HIV care system in St. Louis. IBPA principles were involved at each step to ensure that intersectional principles of power, structure, and social justice were incorporated throughout.

#### Data collection and analysis

We conducted virtual interviews and focus groups via Zoom to identify barriers and facilitators to HIV care, as well as challenges and opportunities for integrating CHWs in the response to HIV in St. Louis. The planning team was responsible for interview materials and participant identification. Interview and focus group guides were designed to address power and positionality through broader questions of perceptions and experiences that were then probed further in the semi-structured interviews. St. Louis participants were identified through professional networks and personal contacts of the planning team to represent different clinical context, expertise, and varied life experiences. We interviewed CHWs and CHW supervisors who participated in a Boston University-led CHW HIV demonstration project (*n* = 6) (26, 30, 43), clinicians (*n* = 4), community leaders (*n* = 5), and people living with HIV (*n* = 12). We also conducted two focus groups (12 participants) with front-line staff working in the field of HIV, such as peer specialists, health coaches, and outreach workers. Demographic information for interview and focus group participants is included in Table 1, showing that participants were predominantly Black American, with a wide range of education and ages. Intersectionality theory informed participant recruiting, as we sought participants from a range of positions concerning HIV services (clients, staff, leadership, community) and from multiple locations, community partners, and diverse positionalities. Interviewers and focus group moderators were inclusively trained, aiming to probe key points such as less socially accepted beliefs and perspectives, and points along the HIV care continuum where different identities influenced the HIV management experience. Analysis and coding were conducted via rapid qualitative analysis methods (68, 69) and mapped to the IBPA framework (52, 57, 70). Interviews were transcribed either internally or by a professional transcription service, with transcripts checked by the interviewers. Each interview or focus group was summarized in a pre-designed template specific to each interviewee category (Appendix A). Templates were completed by one team member based on the interview transcript and then reviewed by the interviewer. The results within participant categories were compiled into separate matrices and summarized within the participant categories (A blank matrix is available in Appendix B). Two researchers developed and approved each summary, and all qualitative team members discussed and agreed on the final synthesized results. Once results were summarized for each participant group, a final matrix synthesizing results across interviewee categories was completed. Similar template categories across interviewee categories were aligned in the matrix, and those results were summarized. The qualitative research team had extensive discussions about interpretations and intersectional implications of participant statements over more than 50 h of virtual meetings from January 2022 through May 2023. Draft findings and interview data were shared with the full planning team, who reviewed conclusions, challenged interpretations, and helped frame all final results.

**Table 1:** Demographic characteristics of community and clinical leaders, frontline health workers, community health workers, and PLWH (*n* = 37).

Characteristic	n (%)
Current gender, n (%)	
Female (cis)	17 (45.9)
Male (cis)	17 (45.9)
Gender-queer or non-binary	1 (2.7)
Trans-woman	2 (5.4)
Race, n (%)	
Black or African American	26 (70.3)
White	9 (24.3)
Not answered	2 (5.4)
Ethnicity, n (%)	
Hispanic or Latino or Spanish origin	0 (0)
Age range, n (%)	
18–25	0
26–35	5 (13.5)
36–45	11 (29.7)
46–55	8 (21.6)
56–65	11 (29.7)
65+	2 (5.4)
Highest level of education completed, n (%)	
No Schooling	1 (2.7)
High School / GED	2 (5.4)
Some college	12 (32.4)
Bachelor’s Degree	4 (10.8)
Masters Degree	11 (29.7)
Associates Degree	4 (10.8)
Professional Degree	1 (2.7)
Not answered	1 (4.0)

Based on the interviews and focus groups, and oriented around the IBPA, a logic model of the problem of engagement and retention in HIV care was developed. Multiple drafts with different visualizations were developed before the planning team realized that a single logic model could not capture the depth of the intersectional dimensions of HIV management, and that two corresponding frameworks were required to embody the non-linearity and complexity of the problem.

We followed the STROBE reporting guidelines, and the checklist is attached as a Supplementary file.

## Results

Results of the intersectional needs assessment are presented through the application of the IBPA, the identified barriers to effective management of HIV, and the problem logic model. We invite readers to bring an intersectional lens to their reading, as space limitations preclude detailed explication throughout.

### IBPA values; planning team values (Q1)

The guiding principles of IBPA include: recognizing the limitations of singular social categories, considering multi-level relationships in analyzing policy impacts, addressing power dynamics across various levels, practicing reflexivity for self-awareness, acknowledging the temporal and spatial dimensions of societal structures, valuing diverse sources and types knowledge, and striving for social justice and equity in policy formulation and analysis (see Table 1). Applications of these guiding principles in the needs assessment and intervention planning are detailed in Table 2. Each guiding principle manifested in multiple ways. For example, the team addressed the issue of power, including the awareness of differential power across care systems and within the planning team, by incorporating a structure for sharing leadership during planning team meetings. Part way through the project two planning team members proposed intentional inclusion of an open ‘unmeeting’^1^ time during the regular biweekly meetings, to encourage all team members to raise questions, additional agenda items, thoughts, and broader systemic concerns. As the initiative progressed the unmeeting space evolved to ensure that everyone had a chance to participate and was eventually stewarded by a community leader and St. Louis native researcher together.

**Table 2:** Applications of the IBPA guiding principles to STEER [adapted from (52); definitions from Humphries et al. (60)].

Guiding principles	Definition and application
Intersecting categories	One social category cannot fully define or explain an individual’s needs and experiences. Intersectionality recognizes that multiple categories underlie each of our lived experience. **Planning team process***: In defining the problem, we took into account the multiple dimensions of identity and lived experience that influence the experiences of PLWH and the* var*ious staff and care providers that work with them, choosing an expansive and inclusive definition of the problem, rather than a more focused and reductionist definition.*
Multi-level analysis	Relationships and associations happen across multiple levels of society and across policies (from the micro to the macro) that can reinforce inequities **Intervention Planning***: In identifying critical components of the response to the problem we have adopted a multilevel approach, including the individual, organizational staff, clinic, community.*
Power	Systems of power have been used across structural levels (local, federal, global) to create and enforce inequities based on identity. IBPA prioritizes recognition of how power can be resisted, replicated, and modified to dismantle systems of inequities. **Planning team process:** *we emphasized relationship building and regular check ins for the planning team. The unmeeting* (see Footnote 1) *provides open space for ongoing discussions, emphasizing the shared responsibility we each have for shaping the agenda, discussion and decisions of the planning team.* **Intervention Planning:** *Our intervention planning is informed by evidence from key stakeholders on the systemic barriers leading to health inequalities and the role of institutions in producing power hierarchies in the healthcare system.*
Reflexivity	Reflexivity reminds researchers, stakeholders, and policy makers to practice self-awareness, recognize positions of privilege, and conduct continual conversations concerning these topics. **Planning team process***: The planning team sought to integrate this principle in our analysis and in our discussions to acknowledge limits to each of our knowledge, various positions of privilege, and to consider insights from applying the intersectionality-based policy analysis*. **Intervention Planning**: *Community members on the research team conducted community co-design sessions, knowing that their positionality over that of the academic researchers, would facilitate reflexive feedback from PLWH and community leaders on intervention plans.*
Time and space	Understanding of the world, societal structures, individuals, and identities are rooted in specific places and times. **Planning team process***: Throughout the analysis we maintained a focus on the situation in St. Louis, acknowledging the role the historical and ongoing racial, economic and social discrimination, disparities and divisions play in the lives of PLWH in St. Louis.*
Diverse knowledges	Recognizing, including and affirming voices and experiences of groups, especially of those that have historically been marginalized, is vital to addressing inequities and dismantling systems of power. **Planning team process:** *Each planning team member is recognized as an expert, some through lived experience, some through education, and all essential for the project. We sought feedback and input from diverse voices through formal interviews, focus groups and community feedback sessions, seeking participation formats that worked for different individuals.* **Intervention Planning:** *We conducted research results checking sessions with young PLWH and trans gender PLWH to ensure their perspectives were captured in results.*
Social justice	Social justice aims to find methods to dismantle inequity in social structures and policies. **Planning team process:** *Application of the guiding principle of social justice encouraged consideration of the intersection of racism, homophobia, sexphobia, poverty, addiction, mental illness, and other factors that drive the HIV epidemic in identifying barriers to HIV care and challenges in implementing the intervention.* **Intervention Planning:** *We prioritized strengthening services for marginalized communities.*
Equity	Equity challenges **everyone** to consider what polices can achieve fairness and justice regardless of privilege and oppression. **Planning team process:** *All planning team members were compensated, either as consultants or as researchers. A brief survey was distributed after each planning team meeting, creating space for feedback and suggestions. Based on planning team feedback midway through the project we integrated an ‘unmeeting’ space in each planning team meeting to allow for unstructured and open feedback and discussion as needed. Application of the guiding principle of equity oriented the planning team to capture changes in equity along with other outcome measurements.* **Intervention Planning***: We compensated the PLWH and front-line health workers who participated in our study the same as the providers and community leaders we interviewed.*

### Application of the IBPA framework to project STEER

Our team emphasized values such as self-awareness, deep listening and reflection, and a commitment to actively engage in changing oneself and systems (Table 3; Q1: *What knowledge, values, and experiences do you bring to this area of analysis?*). In addressing Question 2 (Q2: *What is the ‘problem’ under consideration?*), after extensive discussions over several months, the planning team defined the problem as “structural oppression and systemic barriers create and perpetuate challenges for PLWH in their care management that then lead to inequities in HIV outcomes across intersectional categories.” The problem definition was extensively discussed in multiple planning team meetings until a consensus was reached. The team’s vision was to direct efforts toward systemic and structural changes to better support PLWH. In responding to Q3 we redefined the problem from the PLWH’s perspective, applying an equity lens to our analysis (Table 3; Q3: *How has our representation of the ‘problem’ come about?*). The response to Q4 highlighted how this problem representation differentially affects groups, with a focus on the structural and systemic issues faced by people with low income, people of color, and LGBTQ individuals, all of whom experience inequities in HIV outcomes (Table 3; Q4: *How are groups differentially affected by this representation of the ‘problem’?*). Q5 completes the descriptive part of the analysis by providing examples of current policy responses to HIV in the St. Louis area, such as the efforts of the Ryan White program, the FTC-STL Steering Committee, and other CDC-funded programs for specific target populations (Table 3; Q5: *What are the current policy responses to the ‘problems’?*).

**Table 3:** Application of the intersectionality-based policy analysis (57) framework to Project STEER.

A. Descriptive questions	Responses to descriptive questions
What knowledge, values, and experiences do you bring to this area of analysis?	Knowledge: Structural and systemic challenges / inequality and the impact it has on opportunity and health; Implications of power and positionality; Social work, mental health, substance use, nutrition, infectious diseases; Global and local HIV epidemics; Advocacy and political initiatives around HIV Values: Self-awareness, deep listening and reflection, clarity, understanding, growing; commitment to actively engage in changing self and systems; the power of individuals and the power of hope; utilizing privilege to build more equitable systems and redress historical inequalities; centering the individual, community-centered program design, listening and incorporating feedback to build better systems; passion, showing up for our communities; equity, equality, justice, and truth Experiences: Diverse array of living experiences: metropolitan, rural, urban; diverse array of family situations: Single parents, co-parents, single parent homes, multi-parent homes, small families, large families, recent immigration; diverse array of family expectations around religion, academic environments; experiences with poverty, government assistance, job loss, racism, sexism, gender-ism, abuse; minorities across intersectional categories and as majority group members; experience with healthcare systems as patients and caregivers; dealing with substance abuse and the criminal justice system; examples of how others in our lives have used privilege / power / their voice to help build equality; many prior experiences either living with HIV or working with PLWH
What is the ‘problem’ under consideration?	Structural oppression and systemic barriers create and perpetuate challenges for PLWH in their care management that then lead to disparities in HIV outcomes across intersectional categories.
How has our representation of the ‘problem’ come about?	The planning team representation of the problem evolved as an equity lens was brought to discussion, collaborative interrogation of roles of power in the HIV prevention and treatment space, review of interview data, and a repeated process of revisiting the issue and questions to arrive together at a representation. In striving to orient the problem definition and future solutions from the perspective of PLWH, we have named structural oppression and systemic barriers that perpetuate historic inequities and discrimination.
How are groups differentially affected by this representation of the ‘problem’?	By representing the problem as an issue of structural oppression and systemic barriers differentially impacting individuals living with HIV we are seeking to refocus attention on groups who have been impacted by such systemic and structural issues. This includes people with low income, people of color and gender minorities who have worse outcomes in managing HIV as a chronic disease (see #6 below).
What are the current policy responses to the ‘problems’?	**St. Louis responses: Ryan White programs offer a range of HIV care and support services**: (e.g., Early Intervention Services, Mental Health Services, Substance Abuse Outpatient Care, Food Bank/Home Delivered Meals/, Medical Case Management, Mental Health Education/Risk Reduction, Medical Transportation, Housing, etc.) (1) Challenge: highly siloed response system; restrictive eligibility requirements **Fast Track Cities St. Louis** **CARS** – CDC grant focused on policy change around syphilis, gonorrhea, chlamydia – current grant focused on 18–29 Black men; LGBTQI focus, but general to all Black men
B. Transformative questions	Answers to transformative questions
What inequalities actually exist in relation to the ‘problem’?	People with low income, people of color and gender minorities have worse outcomes in managing HIV as a chronic disease.* Race and ethnicity: African Americans are more likely to be diagnosed with HIV, less likely to continue in care or be virally suppressed in St. Louis and in Missouri as a whole. Gender and sexuality: MSM have higher rates of HIV diagnosis, and lower rates of continuing in care and viral suppression than men and women infected with HIV through heterosexual contact or injection drug use. Age: The age of individuals newly diagnosed with HIV has slightly increased over time, and younger individuals are less likely to engage in HIV care. Socioeconomic status: People living below the poverty line are less likely to be engaged in care, as are people who are homeless or incarcerated. Geography: higher proportion of cases from southeast Missouri, and higher rates of care disparities in central Missouri.
Where and how can (immediate) interventions be made to improve the problems in St. Louis?	Organizations serving people living with HIV need to receive sufficient funds to support clients in meeting basic needs such as food, shelter, transportation, access to services. Re-envision public spaces (such as Juneteenth Park) to create welcoming public social gathering spaces and build community.
What are feasible short, medium and long term solutions?	***Short term:*** Social supports for basic needs Increased availability of CHWs to support PLWH in getting their needs addressed Require state and federal government documentation to show where the need is, and to directly provide resources where the needs are. Increase distribution of support funds through small, front line organizations, while balancing reporting requirements to not over-burden organizations. ***Medium term:*** Actively create community, multidisciplinary and welcoming spaces to hold historic trauma. Ready access to affordable, rapid, highly sensitive home tests Work to ensure continued social and governmental buy-in for socioeconomic supports Integration of HIV services into primary care clinics and practices ***Long term:*** Actively build and integrate healing spaces into health care systems for redressing trauma. Continue vaccine development and testing Transform federal funding organizations to look more like the clients at risk. Strengthen measurement and effective dissemination of research-based information on health disparities to the public across multiple categories (race/ethnicity, class, geographic location, age, etc.). Increase under-represented minority (URM) healthcare workers, to reduce medical mistrust and improve culturally attuned relationships between communities of color and healthcare providers. Prioritize organizations that center race and racism to emphasize the need to dismantle racism to address HIV, reduce emphasis on programs that focus on individual behavior change that aren’t addressing the roots of the drivers of risk. Utilization of a systems thinking lens for framing conversations about the HIV epidemic. Public discussion of interlocking systems of oppression.
How will proposed program reduce inequities?	Developing an effectively networked CHW system to support PLWH will do the following, leading to reductions in inequities. CHWs have been shown to leverage community and social networks to expand reach of policies and impacts and deliver care outside of traditional spaces / within communities, thus improving services to marginalized communities Developing healthcare workers with lived experiences that are from communities that are traditionally under-represented or are part of the highest need areas and strengthening their positions within care teams and care systems will increase availability of culturally competent care. Supporting healthcare workers in new models of care deployment (e.g., funding, supervision, training, community deployment, etc.) will focus on addressing traditional barriers to care that affect communities with greatest care needs Focus on front-line units of care provision will better support patients in managing their own care Promoting cooperation across multiple organizations will help to break down silos, enhance the sharing of information, resources, and best practices, and lead to a more cohesive and effective implementation process.
How will implementation and uptake be assured?	We have actively engaged with community leaders and groups during the intervention development process, such as the Fast Track St. Louis Steering Community, and will present the planned intervention design to multiple local groups for feedback prior to submission of the grant proposal to allow inclusion and revision based on community input.
How will you know if inequalities have been reduced?	Increase in access to medical information (culturally responsive, linguistically appropriate, regardless of urbanicity)—(more reports of info provided in way I can understand, less frustration) Disproportionality of rates of HIV care engagement and VL suppression across racial, ethnic, sexual, gender and age differences will be reduced Increase in populations’ access to care (less reports of not getting what they need) Reductions in stigma (reports of less negative interactions with health care system) Reduction in medical mistrust
How has the process of engaging in an intersectionality-based program analysis transformed: your thinking about relation and structures of power and inequity; the ways in which you and others engage in the work of policy development, implementation and evaluation; broader conceptualizations, relations and effects of power asymmetry in the everyday world	The process of engaging in the IBPA has opened spaces for dialog, learning, and action that has altered power dynamics and fostered social justice. Regarding the program development, implementation, and evaluation, engaging in IBPA has provided deeper insights into the interconnected nature of individuals’ identities. Instead of being a mere sum of their parts, each identity intersects and interacts, creating unique experiences. Overall the IBPA has shifted the conversation dynamically to addressing structural issues with the research process and the proposed intervention.

*Limitations in how St. Louis and Missouri statistics around HIV prevention and treatment are presented preclude more complex intersectional analysis.

The second set of IBPA questions are transformative, bringing other lenses to the problem and encouraging a wider range of questions. We utilized existing publicly available data to identify current inequities, including higher rates of diagnosis and lower rates of care among Black gay and bisexual men, younger individuals, and people living below the poverty line (Q6: *What inequalities actually exist in relation to the ‘problem’?*). The planning team identified potential immediate interventions, such as providing resources to front-line organizations to help PLWH meet basic needs and actively repurposing public spaces to be more welcoming and inclusive (Q7: *Where and how can (immediate) interventions be made to improve the problems in St. Louis?*). The potential immediate interventions are informing the next phases of the STEER project, and particularly the components and implementation strategies of the intervention.

The short, medium, and long-term solutions identified drew upon published literature, the experiences and ideas of key informants, and the collective knowledge and experiences of the planning team, while applying the guiding principles of the IBPA. Short-term solutions focus on meeting basic human needs through social supports, deployment of CHWs, improved alignment of state and federal funds with needs, and increased distribution of support funds through front-line organizations closely connected to communities with greater needs (Q8: *What are feasible short-, medium- and long-term solutions?*). Medium-term solutions include actively creating community spaces to process and heal historic trauma, increasing access to home testing, maintaining the social supports identified in immediate and short-term solutions, and integrating HIV services into primary care settings. These medium-term solutions aim to support individuals by making private and anonymous testing available and increasing the availability of routine care through standard health care channels. Long-term solutions involve addressing historic trauma, continuing biomedical research to develop a vaccine, and undertaking multipronged efforts to change the priorities and approaches of funding organizations, strengthening multidimensional measurement of health inequities, increasing representation of minorities in healthcare professions, and prioritizing work and funding through organizations that prioritize responding to impacts of race and racism more than emphasis on individual behavior change.

The next three questions address how the program will reduce inequities, ensure implementation and uptake, and measure changes in inequalities. We expect the program will most immediately reduce inequities by enhancing organizational capacity to integrate CHWs with lived experience from the local community, training them, innovating care team implementation, and promoting cooperation across multiple organizations (Q9: *How will proposed program reduce inequities?*). Regarding implementation and uptake, we have engaged with community leaders and groups through key informant interviews and other outreach activities to gather feedback, ideas and input on the intervention design and implementation strategies (Q10: *How will implementation and uptake be assured?*). Demonstrating that inequalities have been reduced will involve process measures (such as the availability of culturally appropriate medical information), outcome measures (such as reductions in inequities of HIV care engagement and viral suppression), and longer-term systemic change measures (such as reductions in stigma and medical mistrust) (Q11: *How will you know if inequalities have been reduced?*).

The final question of the IBPA reflects on the process, with planning team members noting that the IBPA framework facilitated dialog and learning that shifted power dynamics and promoted social justice (Q12). Utilizing the IBPA led to deeper engagement and understanding of interlocking issues and identities, focused the conversation on structural and systemic issues within both the research process and the planned intervention (Q12: *How has the process of engaging in an intersectionality-based program analysis transformed: your thinking about relation and structures of power and inequity; the ways in which you and others engage in the work of policy development, implementation and evaluation; broader conceptualizations, relations and effects of power asymmetry in the everyday world*).

### Barriers to effective management of HIV for PLWH

**Figure 2:**
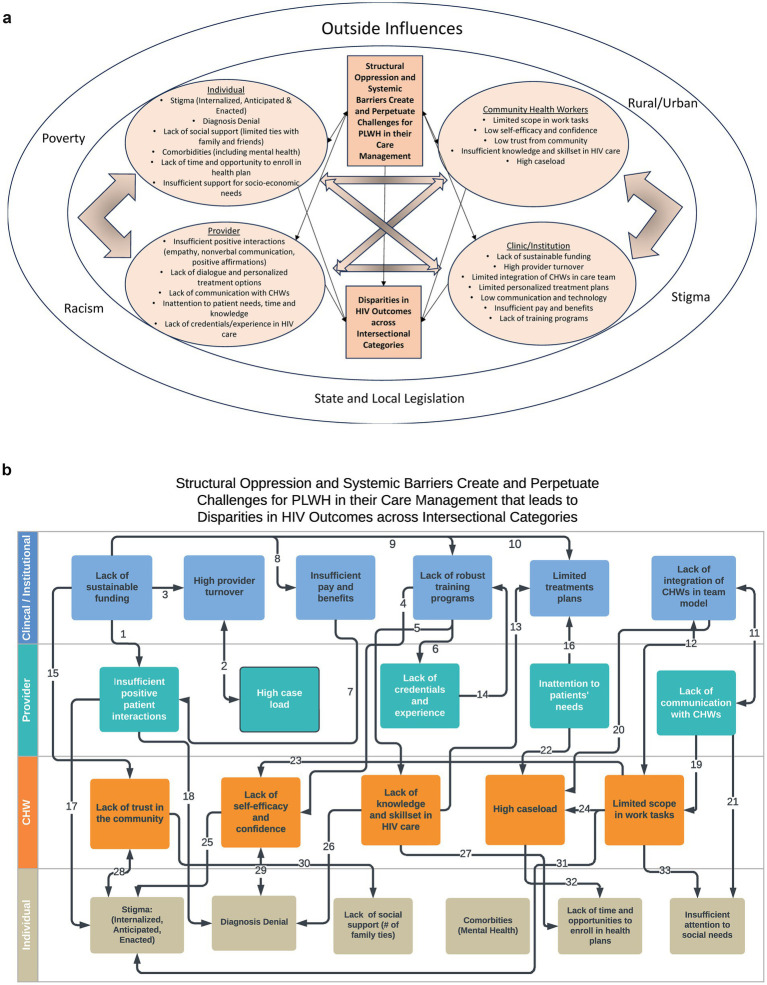
**(a)** Nodal model showing systemic and contextual influences on problem. **(b)** Linear model of problem pathways based on relevant literature, interviews and focus groups.

Across key informants, barriers to care for PLWH were identified at multiple levels, including the clinic/institutional level, provider level, CHW level and individual level (see Figure 2).

#### Clinic/institutional barriers

High provider turnover, lack of sustainable funding for CHWs and other front-line staff, insufficient pay and benefits for CHWs and front-line staff, limited integration of CHWs into care teams, and limited organizational capacity for personalized treatment plans were all identified as barriers to effective management of HIV.

*“[W]e saw a great turnover with the providers at this office, like if you look back over the last eight years there’s probably been 12 providers that have worked here, so people’s level of engagement often times was quite low and their follow through. So as clinicians picked up patients, they did not really have a good handle on the patient’s history, both from a social perspective or medical perspective… so I think if I put myself in a patient’s situation, I would have over time like decreased interest in my health care if I’m constantly experiencing clinician turnover. And I think because providers were here for such a short period of time they only touch on very superficial issues as they develop rapport with the patients, so like the deeper issues that affect their long-term care, I never really addressed.” (*62–66) [Clinical Lead #2].

Adequate compensation for CHWs and similar front-line staff, sustainability of funding, and availability of comprehensive training programs were noted as significant factors impacting the organizational ability to provide effective assistance to PLWH. As one community leader noted,

*“Most of our CHWs, and this is a nationwide thing, are supplied by grants, which means that their scope of service is then indicated by what that grant will allow, but also they do not have long-term viability for their career…. [What] I’ve learned is that the region, the state, and the nation value them, but it’s short-lived when it comes to the operationality as well as the true infrastructure to support those workforces.” [130–133] [Community Lead #5].*

#### Provider level barriers

Provider barriers that impact HIV management include challenges such as high workloads and limited time for providers to meet with each patient, as well as issues arising from provider knowledge and behaviors, such as limited awareness of CHWs and other front-line staff. Clinical leaders reported being burdened with multiple responsibilities and having limited capacity to support clients in need.

*I think most physicians that I work with… have a lot of other responsibilities, and they’ll see patients every two, three months. If people are doing less well, they might see him every three or four weeks. But I certainly think, and I have an expectation that if someone’s really struggling, a health coach is someone that could … contact the patient once or twice a week. I’ve had some health coaches that text patients every day to remind them to take their meds …This is something I certainly could not do. [Clinical Lead #4].*

In addition, clinical leaders noted challenges in understanding the responsibilities of new types of support staff as innovative roles are introduced to the team.

*[O]ne of the problems with … Ryan White funded clinics, is that it provides money for a lot of type of roles like a health coach, but as soon as you get beyond the minimal office staff of your front desk person who handles taking in patients, and your medical assistants, the nurse, and the doctor, [nurse practitioner], you start adding people and you add people with more finely described roles. I think getting that information out so people understand who these people are and what they can offer is not easy…. [Clinical Lead #4].*

#### CHW and front-line staff barriers

Similar to clinical providers, CHWs and other front-line staff encounter their own set of challenges that impact the care they provide to PLWH. These challenges include limited trust from the community, a lack of self-efficacy and confidence, insufficient knowledge and skills related to HIV, high caseloads, and scope of work limited by organizations.

*“[T]he distrust and the disconnect between medical care is so vast, if you come with a prescriptive posture it’s over before it starts, you have to do something different.” [Clinical Lead #1].*

Front-line health workers highlighted the importance of dedicating time to earn their clients’ trust.

*“[Y]ou have to win their trust before you can test them [for HIV] because you have to … earn their trust, so they can open up and you can talk…. And when they trust you they can open up and they’ll talk to you, and you can have that one on one relationship where you can say hey let us try this and then we talk in a week you let me know how you like that, and if you do not like that then we’ll try something else.” [FG1-Prevention Specialist].*

Front-line health workers also noted the importance of having time to discuss various approaches and the necessity of offering training and skill-building opportunities.

*“I think we did more home visits back then, and we were able to spend a little more time with our clients doing some skill training and stuff like that. I think now we are so focused on trying to get progress notes done that we are not be able, you know we got so many clients that you cannot spend as much time with them as you used to.” [FG2-Supervisor].*

#### PLWH barriers

Participants from all stakeholder groups identified challenges that impact the ability of PLWH to manage their HIV. Key themes included racial stigma, diagnosis denial, lack of social support, comorbidities, medication side effects, lack of time and opportunity to access health plans, and the inability to address basic needs.

*“There’s always going to be a stigma attached to being HIV positive, and I think part of that is in the African American community, and I think the community health workers would have to ask that population why they drop out of care to actually get a real sense of why they are not staying in care and take their meds, whether it be the other things that we have discussed already or if there’s some other reason why they are not in care or staying in care.” [Clinical Lead 3].*

Addressing comorbidities such as mental health and chronic disease as well as challenges in meeting basic needs is crucial for supporting effective management of HIV by PLWH.

*“We try to help people stay in care, but sometimes our clients have severe and persistent mental illnesses, they are not ready to be in care so sometimes they do not follow up with services, it’s too regimented. So we kind of leave them on the fringe, but we still talk to them and try to say, well, when you need me call me…And so six months down the road they are like … I need emergency housing, let me call [participant’s name].… So we get them back into care. (FG2-Supervisor).”*

PLWH need access to a range of services and may have multiple specialist care needs.

*“[W]ith our clients and patients that we work with you know, besides going to their medical appointments, dental appointments, and the other specialist appointments they might have, they do not have transportation, you know, they do not feel well, you know they are in a really difficult spot in their life and they feel alone.…” (FG1-Health Coach2).*

### Logic model of the problem

Drawing on the results of the needs assessment and the HIV literature the planning team developed two models of the problem (Figures 2a,b), each highlighting different but integrated dimensions. The team determined both versions of the problem logic model were necessary to fully incorporate the complex intersectional factors that influence the success or failure of future interventions. The first model (Figure 2a) adopts a nodal approach, emphasizing the mutually reinforcing challenges that affect management of HIV. The second model (Figure 2b) employs a linear approach, focusing on the pathways of influence between various barriers to HIV management.

In Figure 2a, the team positioned CHWs in a distinct node to draw attention to their role as intermediaries between institutions and individuals, while also highlighting their unique resource constraints. Although structural oppression and systemic barriers were specifically mentioned in the statement of the problem, Figure 2a also acknowledges that poverty, racism, stigma, legislation and rural/urban issues are important influences.

In Figure 2b, the arrows illustrate the multiple pathways through which clinical and institutional challenges impact providers (arrows 1, 2, 6 and 11) and CHWs (arrows 4, 5, 12 and 15), as well as the pathways by which challenges at the provider and CHW level impact clients. Arrows were identified from the literature and in discussions of the planning team. Each pathway represents potential places where multiple identities and/or positionalities influence structural outcomes. Supporting documentation for the arrows in Figure 2b is provided in Appendix C.

## Discussion

This study conducted an innovative participatory intersectional needs assessment to facilitate the adaptation and implementation planning of an intervention integrating CHWs into the HIV care system. While prior applications of the IBPA have focused on policy change (52, 71), this project represents the first use to our knowledge of the IBPA as a framework for conducting a participatory needs assessment. The IBPA fostered a focus on intersectional challenges, ensuring we paid explicit attention to less heard from voices. A recent meta-analysis on stigma and intersectionality further supports the approach, showing that intersectionality deepens the understanding of stigma experienced across social identities (72).

During the IBPA process the research team encountered several challenges, including ensuring that both community and academic partners understood the IBPA and its role in informing the research. We spent significant time during planning team meetings presenting, discussing and unpacking how we were going to use the IBPA in our work, and then reviewing and discussing examples of applying the guiding principles and responses to all of the IBPA questions. Balancing the need to complete the IBPA process with the priority of reviewing qualitative and quantitative documents in a timely manner required allocating purposeful time in meetings and creating planning team meeting slide decks and notes, as well as sharing draft versions of the IBPA tables to track themes and discussions. Valuing the expertise while navigating the priorities of community partners without overburdening them, required careful planning.

Barriers to effective HIV management for PLWH, consistent with existing literature, were identified at the clinic/institutional, provider, CHW and client levels. Specific barriers reported at the clinic/institutional level included high turnover, insufficient funding for CHWs, limited integration of CHWs in care teams, and restricted organizational capacity for personalized treatment. Provider barriers reported encompassed high workloads, limited time, and inadequate awareness of CHWs skills. CHWs and front-line staff reported facing challenges, including community distrust, low self-efficacy and confidence, insufficient HIV knowledge and skills, high caseloads, and a restricted scope of work. Client-level barriers reported included stigma, diagnosis denial, lack of social support, co-morbidities and medication side effects, healthcare access difficulties, and unmet basic needs. We have reported previously on co-morbidities of mental health and substance use (73). Previous studies have identified similar client-level barriers to HIV treatment engagement, including competing life activities, poor transportation, stigma, lack of insurance coverage, poverty, and beliefs about HIV care (74–83). Among Black women living with HIV, HIV-related stigmas expressed by healthcare providers to them and poor quality HIV services have been identified as key barriers to treatment engagement (74, 84, 85). At the organizational level, although research is limited, studies have identified challenges such as finding providers who speak the same language as patients and treat them with respect, providing team-based care, managing the cost of services, and navigating the logistics of the care system (86, 87). Previously identified clinic and provider-level facilitators include patient friendly services and positive, trusting relationships with providers (77, 88, 89). We are reporting separately on the barriers to integrating CHWs and implementation strategies to address those barriers (manuscript in preparation).

The logic model of the problem highlighted intersectional issues by utilizing two complementary formats: a nodal model and a linear pathways model, to capture the intersectional and systemic issues. While problem logic models typically offer a focused representation of a problem’s components, the planning team found it challenging to agree on a singular linear model. Utilizing two models emphasizes the importance of both the systemic drivers and the specific pathways where positionality and intersectional dynamics are enacted.

We report here on the initial steps of the IntMap and ImpMap protocols, with the needs assessment results intended to inform the future adaptation of the CHW approach. This is an important contribution to the growing field of equity-centered implementation within the area of HIV and more broadly (90, 91). Results identifying implementation strategies and the development of an implementation plan to address identified barriers and enhance implementation outcomes (92–95) will be presented elsewhere.

### Limitations

This study was centered on St. Louis, and while its processes are relevant to other settings, specific barriers require validation in other populations. Due to challenges in recruiting younger PLWH and transgender individuals for interviews, future activities will include structured community conversations at St. Louis locations frequented by these groups to gather their input.

## Conclusion

Use of an intersectional framework contributed to identification of systemic and structural barriers to effective HIV management. The systemic and structural perspective was also apparent in the two complementary logic models of the problem developed by the team. Barriers to effective HIV management were identified across multiple levels, including organizations, providers, CHWs, front-line staff and clients. Emphasizing an intersectional approach highlighted similar barriers as previous research to HIV management at the individual, provider and front-line staff levels. At the clinic and institutional level additional barriers were identified such as high staff turnover, insufficient CHWs and insufficient CHW integration. This participatory intersectional needs assessment has identified local challenges and priorities to be addressed in the adapted intervention and implementation strategies.

## Data Availability

The raw data supporting the conclusions of this article will be made available by the authors, without undue reservation.

## Footnotes

^1^An unmeeting is an intentional creation of an unstructured space for informal discussions without the rules, format or constraints of a formal meeting.

## References

[1] Fauci AS Redfield RR Sigounas G Weahkee MD Giroir BP. Ending the HIV epidemic: a plan for the United States. JAMA. (2019) 321:844–5. doi: 10.1001/jama.2019.1343, PMID: 30730529

[2] Giroir BP. The time is now to end the HIV epidemic. Am J Public Health. (2020) 110:22–4. doi: 10.2105/AJPH.2019.305380, PMID: 31725312 PMC6893354

[3] Nosyk B Zang X Krebs E Enns B Min JE Behrends CN. Ending the HIV epidemic in the USA: an economic modelling study in six cities. Lancet HIV. (2020) 7:e491–503. doi: 10.1016/S2352-3018(20)30033-3, PMID: 32145760 PMC7338235

[4] Centers for Disease Control and Prevention. Estimated HIV incidence and prevalence in the United States, 2017–2021: Centers for Disease Control and Prevention; (2023). Available online at: http://www.cdc.gov/hiv/library/reports/hiv-surveillance.html.

[5] Bowleg L. The problem with the phrase women and minorities: intersectionality-an important theoretical framework for public health. Am J Public Health. (2012) 102:1267–73. doi: 10.2105/AJPH.2012.300750, PMID: 22594719 PMC3477987

[6] Chenneville T Gabbidon K Brinson A Capobianco J Cario A Rodriguez C. Lived experiences of racism and intersectional stigma among black youth living with HIV in the deep south. Stigma Health. (2022) 8:299–314.

[7] Quinn KG. Applying an intersectional framework to understand syndemic conditions among young black gay, bisexual, and other men who have sex with men. Soc Sci Med. (2022) 295:112779. doi: 10.1016/j.socscimed.2019.112779, PMID: 31898991 PMC7319868

[8] Missouri Department of Health and Senior Services. Ending the HIV epidemic (EHE) (2021). Available online at: https://health.mo.gov/living/healthcondiseases/communicable/hivaids/pdf/ending-draft-plan.pdf (Accessed April 03, 2022).

[9] Baugher AR Whiteman A Jeffries WL Finlayson T Lewis R Wejnert C. Black men who have sex with men living in states with HIV criminalization laws report high stigma, 23 U.S. cities, 2017. AIDS. (2021) 35:1637–45. doi: 10.1097/QAD.0000000000002917, PMID: 34270489 PMC9030111

[10] Quinn KG John SA Hirshfield S Algiers O O'Neil A Petroll AE. Challenges to meeting the HIV care needs of older adults in the rural south. SSM - Qualitative Res Health. (2022) 2:100113. doi: 10.1016/j.ssmqr.2022.100113, PMID: 36620181 PMC9815493

[11] Gilbert KL Ray R Siddiqi A Shetty S Baker EA Elder K. Visible and invisible trends in black men's health: pitfalls and promises for addressing racial, ethnic, and gender inequities in health. Annu Rev Public Health. (2016) 37:295–311. doi: 10.1146/annurev-publhealth-032315-021556, PMID: 26989830 PMC6531286

[12] Ingram M Doubleday K Bell ML Lohr A Murrieta L Velasco M. Community health worker impact on chronic disease outcomes within primary care examined using electronic health records. Am J Public Health. (2017) 107:1668–74. doi: 10.2105/AJPH.2017.303934, PMID: 28817321 PMC5607666

[13] Patel MI Kapphahn K Dewland M Aguilar V Sanchez B Sisay E. Effect of a community health worker intervention on acute care use, advance care planning, and patient-reported outcomes among adults with advanced stages of cancer: a randomized clinical trial. JAMA Oncol. (2022) 8:1139–48. doi: 10.1001/jamaoncol.2022.1997, PMID: 35771552 PMC9247857

[14] Perry HB Zulliger R Rogers MM. Community health workers in low-, middle-, and high-income countries: an overview of their history, recent evolution, and current effectiveness. Annu Rev Public Health. (2014) 35:399–421. doi: 10.1146/annurev-publhealth-032013-182354, PMID: 24387091

[15] Islam N Nadkarni SK Zahn D Skillman M Kwon SC Trinh-Shevrin C. Integrating community health workers within patient protection and affordable care act implementation. J Public Health Manag Pract. (2015) 21:42–50. doi: 10.1097/PHH.0000000000000084, PMID: 25414955 PMC4416641

[16] Daniels AS Bergeson S Myrick KJ. Defining peer roles and status among community health workers and peer support specialists in integrated Systems of Care. Psychiatr Serv. (2017) 68:1296–8. doi: 10.1176/appi.ps.201600378, PMID: 28712350

[17] Pinto D Carroll-Scott A Christmas T Heidig M Turchi R. Community health workers: improving population health through integration into healthcare systems. Curr Opin Pediatr. (2020) 32:674–82. doi: 10.1097/MOP.0000000000000940, PMID: 32889962

[18] Mistry SK Harris E Harris M. Community health workers as healthcare navigators in primary care chronic disease management: a systematic review. J Gen Intern Med. (2021) 36:2755–71. doi: 10.1007/s11606-021-06667-y, PMID: 33674916 PMC8390732

[19] Kamanda A Embleton L Ayuku D Atwoli L Gisore P Ayaya S. Harnessing the power of the grassroots to conduct public health research in sub-Saharan Africa: a case study from western Kenya in the adaptation of community-based participatory research (CBPR) approaches. BMC Public Health. (2013) 13:1–10. doi: 10.1186/1471-2458-13-91, PMID: 23368931 PMC3564692

[20] Peretz PJ Islam N Matiz LA. Community health workers and Covid-19 - addressing social determinants of health in times of crisis and beyond. N Engl J Med. (2020) 383:e108. doi: 10.1056/NEJMp2022641, PMID: 32966715

[21] Wolfe HL Baughman A Davoust M Sprague Martinez LS Rajabiun S Drainoni ML. Client satisfaction with community health workers in HIV care teams. J Community Health. (2021) 46:951–9. doi: 10.1007/s10900-021-00978-1, PMID: 33770333

[22] Davoust M Drainoni M-L Baughman A Campos Rojo M Estes T Rajabiun S. “He gave me Spirit and Hope”: client experiences with the implementation of community health worker programs in HIV care. AIDS Patient Care STDs. (2021) 35:318–26. doi: 10.1089/apc.2021.0085, PMID: 34375140 PMC8380790

[23] Kenya S Jones J Arheart K Kobetz E Chida N Baer S. Using community health workers to improve clinical outcomes among people living with HIV: a randomized controlled trial. AIDS Behav. (2013) 17:2927–34. doi: 10.1007/s10461-013-0440-1, PMID: 23515640 PMC4184095

[24] Kenya S Chida N Symes S Shor-Posner G. Can community health workers improve adherence to highly active antiretroviral therapy in the USA? A review of the literature. HIV Med. (2011) 12:525–34. doi: 10.1111/j.1468-1293.2011.00921.x, PMID: 21518221

[25] Shaghaghi A Bhopal RS Sheikh A. Approaches to recruiting 'hard-to-reach' populations into re-search: a review of the literature. Health Promot Perspect. (2011) 1:86–94. doi: 10.5681/hpp.2011.009, PMID: 24688904 PMC3963617

[26] Sprague Martinez L Davoust M Rajabiun S Baughman A Bachman SS Bowers-Sword R. "part of getting to where we are is because we have been open to change" integrating community health workers on care teams at ten Ryan white HIV/AIDS program recipient sites. BMC Public Health. (2021) 21:922. doi: 10.1186/s12889-021-10943-1, PMID: 33990190 PMC8120741

[27] Kangovi S Mitra N Grande D Long JA Asch DA. Evidence-based community health worker program addresses unmet social needs and generates positive return on investment. Health Aff (Millwood). (2020) 39:207–13. doi: 10.1377/hlthaff.2019.00981, PMID: 32011942 PMC8564553

[28] Acharya H Sykes KJ Neira TM Scott A Pacheco CM Sanner M. A novel electronic record system for documentation and efficient workflow for community health workers: development and usability study. JMIR Form Res. (2024) 8:e52920. doi: 10.2196/52920, PMID: 38557671 PMC11019415

[29] Kok MC Dieleman M Taegtmeyer M Broerse JE Kane SS Ormel H. Which intervention design factors influence performance of community health workers in low- and middle-income countries? A systematic review. Health Policy Plan. (2015) 30:1207–27. doi: 10.1093/heapol/czu126, PMID: 25500559 PMC4597042

[30] Drainoni M-L Baughman AL Bachman SS Bowers-Sword R Davoust M Fortu K. Integrating community health workers into HIV care teams: impact on HIV care outcomes. J HIV AIDS Soc Serv. (2020) 19:204–19. doi: 10.1080/15381501.2020.1785364

[31] Fernandez ME Ten Hoor GA van Lieshout S Rodriguez SA Beidas RS Parcel G. Implementation mapping: using intervention mapping to develop implementation strategies. Front Public Health. (2019) 7:158. doi: 10.3389/fpubh.2019.00158, PMID: 31275915 PMC6592155

[32] Bartholomew LK Parcel GS Kok G. Intervention mapping: a process for developing theory- and evidence-based health education programs. Health Educ Behav. (1998) 25:545–63. 9768376 10.1177/109019819802500502

[33] Eldredge LKB Markham CM Ruiter RA Fernández ME Kok G Parcel GS. Planning health promotion programs: An intervention mapping approach. Hoboken, NJ, USA: John Wiley & Sons (2016).

[34] Bauer MS Damschroder L Hagedorn H Smith J Kilbourne AM. An introduction to implementation science for the non-specialist. BMC Psychol. (2015) 3:32. doi: 10.1186/s40359-015-0089-9, PMID: 26376626 PMC4573926

[35] Leeman J Birken SA Powell BJ Rohweder C Shea CM. Beyond "implementation strategies": classifying the full range of strategies used in implementation science and practice. Implement Sci. (2017) 12:125. doi: 10.1186/s13012-017-0657-x, PMID: 29100551 PMC5670723

[36] Damschroder LJ. Clarity out of chaos: use of theory in implementation research. Psychiatry Res. (2020) 283:112461. doi: 10.1016/j.psychres.2019.06.036, PMID: 31257020

[37] Center for Community Health and Development. Community tool box Kansas: university of Kansas; 1994–2023 [community-building skills tool box]. (2023). Available online at: https://ctb.ku.edu/en/table-of-contents/analyze/where-to-start/participatory-approaches/main (Accessed September 18, 2023).

[38] Suen JJ Marrone N Han HR Lin FR Nieman CL. Translating public health practices: community-based approaches for addressing hearing health care disparities. Semin Hear. (2019) 40:37–48. doi: 10.1055/s-0038-1676782, PMID: 30728648 PMC6363549

[39] Yang KI Chung-Do JJ Fujitani L Foster A Mark S Okada Y. Advancing community-based participatory research to address health disparities in Hawai'i: perspectives from academic researchers. Hawaii J Med Public Health. (2019) 78:83–8. PMID: 30854253 PMC6401203

[40] Burgess R Dedios Sanguineti MC Maldonado-Carrizosa D Fonseca L Vera San Juan N Lucumi D. Using participatory action research to reimagine community mental health services in Colombia: a mixed-method study protocol. BMJ Open. (2022) 12:e069329. doi: 10.1136/bmjopen-2022-069329, PMID: 36549743 PMC9772630

[41] Douglas JA Subica AM Franks L Johnson G Leon C Villanueva S. Using participatory mapping to diagnose upstream determinants of health and prescribe downstream policy-based interventions. Prev Chronic Dis. (2020) 17:E138. doi: 10.5888/pcd17.200123 33155972 PMC7665598

[42] Mann-Jackson L Alonzo J Garcia M Trent S Bell J Horridge DN. Using community-based participatory research to address STI/HIV disparities and social determinants of health among young GBMSM and transgender women of colour in North Carolina, USA. Health Soc Care Community. (2021) 29:e192–203. doi: 10.1111/hsc.13268, PMID: 33369811 PMC8451894

[43] Rajabiun S Baughman A Sullivan M Poteet B Downes A Davich JAW. A participatory curricula for community health workers and supervisors to increase HIV health outcomes. Front Public Health. (2021) 9:689798. doi: 10.3389/fpubh.2021.689798 34395367 PMC8362906

[44] Rhodes SD Kelley C Simán F Cashman R Alonzo J McGuire J. Using community-based participatory research (CBPR) to develop a community-level HIV prevention intervention for Latinas: a local response to a global challenge. Womens Health Issues. (2012) 22:e293–301. doi: 10.1016/j.whi.2012.02.002, PMID: 22483581 PMC3627530

[45] Mwamba C Beres LK Mukamba N Jere L Foloko M Lumbo K. Provider perspectives on patient-centredness: participatory formative research and rapid analysis methods to inform the design and implementation of a facility-based HIV care improvement intervention in Zambia. J Int AIDS Soc. (2023) 26:e26114. doi: 10.1002/jia2.26114, PMID: 37408458 PMC10323320

[46] Karris MY Dube K Moore AA. What lessons it might teach us? Community engagement in HIV research. Curr Opin HIV AIDS. (2020) 15:142–9. doi: 10.1097/COH.0000000000000605, PMID: 31895141 PMC7374765

[47] Valdiserri RO Holtgrave DR. Ending HIV in America: not without the power of community. AIDS Behav. (2019) 23:2899–903. doi: 10.1007/s10461-019-02496-7, PMID: 30953303

[48] Anonymous. The Denver Principles. (1983). Available online at: https://data.unaids.org/pub/externaldocument/2007/gipa1983denverprinciples_en.pdf (Accessed August 30, 2024).

[49] Holliday RC Phillips R Akintobi TH. A community-based participatory approach to the development and implementation of an HIV health behavior intervention: lessons learned in navigating research and practice systems from project HAPPY. Int J Environ Res Public Health. (2020) 17:399. doi: 10.3390/ijerph17020399, PMID: 31936190 PMC7014096

[50] Rajabiun S Lennon-Dearing R Hirschi M Davis B Williams B Sprague Martinez L. Ending the HIV epidemic: one southern community speaks. Soc Work Public Health. (2021) 36:647–64. doi: 10.1080/19371918.2021.1947929, PMID: 34251984 PMC8429130

[51] Oliver B Pearl C John EF Norris D Olaniyan FE Samson K. Understanding lived experiences of stigma for people living with HIV: a community based participatory research study. Qual Rep. (2023) 28:623–43. doi: 10.46743/2160-3715/2023.528

[52] Hankivsky O. An intersectionality-based policy analysis framework. Vancouver, BC: Institute for Intersectionality Research and Policy, Simon Fraser University (2012).

[53] Kapilashrami A Hankivsky O. Intersectionality and why it matters to global health. Lancet. (2018) 391:2589–91. doi: 10.1016/S0140-6736(18)31431-4, PMID: 30070211

[54] Collins PH. Black feminist thought: Knowledge, consciousness, and the politics of empowerment. Oxfordshire, England, UK: Routledge (2022).

[55] Crenshaw K. Demarginalizing the intersection of race and sex: a black feminist critique of antidiscrimination doctrine, feminist theory and antiracist politics. Univ Chicago Legal F. (1989) 1:139.

[56] Watkins-Hayes C. Intersectionality and the sociology of HIV/AIDS: past, present, and future research directions. Annu Rev Sociol. (2014) 40:431–57. doi: 10.1146/annurev-soc-071312-145621

[57] Hankivsky O Grace D Hunting G Giesbrecht M Fridkin A Rudrum S. An intersectionality-based policy analysis framework: critical reflections on a methodology for advancing equity. Int J Equity Health. (2014) 13:119. doi: 10.1186/s12939-014-0119-x, PMID: 25492385 PMC4271465

[58] Hankivsky O. Women's health, men's health, and gender and health: implications of intersectionality. Soc Sci Med. (2012) 74:1712–20. doi: 10.1016/j.socscimed.2011.11.029, PMID: 22361090

[59] Hankivsky O Reid C Cormier R Varcoe C Clark N Benoit C. Exploring the promises of intersectionality for advancing women's health research. Int J Equity Health. (2010) 9:5. doi: 10.1186/1475-9276-9-5, PMID: 20181225 PMC2830995

[60] Humphries DL Sodipo M Jackson SD. The intersectionality-based policy analysis framework: demonstrating utility through application to the pre-vaccine U.S. COVID-19 policy response. Front Public Health. (2023) 11:1040851. doi: 10.3389/fpubh.2023.1040851 37655290 PMC10466398

[61] Bowleg L. Evolving intersectionality within public health: from analysis to action. Am J Public Health. (2021) 111:88–90. doi: 10.2105/AJPH.2020.306031, PMID: 33326269 PMC7750585

[62] McBride K Carlson M Everett B. Using the intersectionality-based policy analysis framework to evaluate a policy supporting sexual health and intimacy in long-term care, assisted living, group homes & supported housing. J Appl Gerontol. (2022) 41:1992–2001. doi: 10.1177/07334648221099728 35623344

[63] Isiwele A Rivas C Stokes G. Nigerian and Ghanaian young people's experiences of Care for Common Mental Disorders in inner London: protocol for a multimethod investigation. JMIR Res Protoc. (2022) 11:e42575. doi: 10.2196/42575, PMID: 36485025 PMC9789493

[64] Kehoe MacLeod K Flores KN Chandra K. Identifying facilitators and barriers to integrated and equitable care for community-dwelling older adults with high emergency department use from historically marginalized groups. Int J Equity Health. (2023) 22:97. doi: 10.1186/s12939-023-01900-y, PMID: 37208757 PMC10198019

[65] Fernandez ME Ruiter RAC Markham CM Kok G. Intervention mapping: theory- and evidence-based health promotion program planning: perspective and examples. Front Public Health. (2019) 7:209. doi: 10.3389/fpubh.2019.00209, PMID: 31475126 PMC6702459

[66] Merz S Jaehn P Mena E Pöge K Strasser S Saß A-C. Intersectionality and eco-social theory: a review of potentials for public health knowledge and social justice. Crit Public Health. (2023) 33:125–34. doi: 10.1080/09581596.2021.1951668

[67] Fast-track cities. Fast-Track Cities Global Web Portal (2021). Available online at: https://fast-trackcities.org/about (Accessed April 15, 2021).

[68] McNall M Foster-Fishman PG. Methods of rapid evaluation, assessment, and appraisal. J American J Evaluation. (2007) 28:151–68. doi: 10.1177/1098214007300895

[69] Vindrola-Padros C Johnson GA. Rapid techniques in qualitative research: a critical review of the literature. Qual Health Res. (2020) 30:1596–604. doi: 10.1177/1049732320921835, PMID: 32667277

[70] Damschroder LJ Reardon CM Widerquist MAO Lowery J. The updated consolidated framework for implementation research based on user feedback. Implement Sci. (2022) 17:75. doi: 10.1186/s13012-022-01245-0, PMID: 36309746 PMC9617234

[71] Hankivsky O Cormier R. Intersectionality and public policy: some lessons from existing models. Polit Res Q. (2011) 64:217–29. doi: 10.1177/1065912910376385

[72] Jackson-Best F Edwards N. Stigma and intersectionality: a systematic review of systematic reviews across HIV/AIDS, mental illness, and physical disability. BMC Public Health. (2018) 18:919. doi: 10.1186/s12889-018-5861-3, PMID: 30049270 PMC6062983

[73] Marotta PL Carter CR Hu Y Wang V Rucker D Jones J. “They need people to actually show up in their lives:” deploying community health workers to improve mental health and substance use outcomes among PLWH. J HIV AIDS Soc Serv. (2024) 22:1–23. doi: 10.1080/15381501.2024.2385988

[74] Algarin AB Sheehan DM Varas-Diaz N Fennie KP Zhou Z Spencer EC. Health care-specific enacted HIV-related stigma's association with antiretroviral therapy adherence and viral suppression among people living with HIV in Florida. AIDS Patient Care STDs. (2020) 34:316–26. doi: 10.1089/apc.2020.0031, PMID: 32639208 PMC7370977

[75] Chang EJ Fleming M Nunez A Dombrowski JC. Predictors of successful HIV care re-engagement among persons poorly engaged in HIV care. AIDS Behav. (2019) 23:2490–7. doi: 10.1007/s10461-019-02491-y, PMID: 30980279

[76] Krentz HB McPhee P Arbess G Jackson L Stewart AM Bois D. Reporting on patients living with HIV "disengaging from care". Who is actually "lost to follow-up"? AIDS Care. (2021) 33:114–20. doi: 10.1080/09540121.2020.1761516, PMID: 32408758

[77] Yehia BR Stewart L Momplaisir F Mody A Holtzman CW Jacobs LM. Barriers and facilitators to patient retention in HIV care. BMC Infect Dis. (2015) 15:246. doi: 10.1186/s12879-015-0990-0, PMID: 26123158 PMC4485864

[78] Bulsara SM Wainberg ML Newton-John TRO. Predictors of adult retention in HIV care: a systematic review. AIDS Behav. (2018) 22:752–64. doi: 10.1007/s10461-016-1644-y, PMID: 27990582 PMC5476508

[79] Sheehan DM Fennie KP Mauck DE Maddox LM Lieb S Trepka MJ. Retention in HIV care and viral suppression: individual- and neighborhood-level predictors of racial/ethnic differences, Florida, 2015. AIDS Patient Care STDs. (2017) 31:167–75. doi: 10.1089/apc.2016.0197, PMID: 28414260 PMC5397217

[80] Hall BJ Sou KL Beanland R Lacky M Tso LS Ma Q. Barriers and facilitators to interventions improving retention in HIV care: a qualitative evidence Meta-synthesis. AIDS Behav. (2017) 21:1755–67. doi: 10.1007/s10461-016-1537-0, PMID: 27582088 PMC5332336

[81] Lee H Wu XK Genberg BL Mugavero MJ Cole SR Lau B. Beyond binary retention in HIV care: predictors of the dynamic processes of patient engagement, disengagement, and re-entry into care in a US clinical cohort. AIDS. (2018) 32:2217–25. doi: 10.1097/QAD.0000000000001936, PMID: 30005018 PMC6136972

[82] Nijhawan AE Liang Y Vysyaraju K Munoz J Ketchum N Saber J. Missed initial medical visits: predictors, timing, and implications for retention in HIV care. AIDS Patient Care STDs. (2017) 31:213–21. doi: 10.1089/apc.2017.0030 28488891 PMC5485218

[83] Swann M. Economic strengthening for HIV prevention and risk reduction: a review of the evidence. AIDS Care. (2018) 30:37–84. doi: 10.1080/09540121.2018.1479029 29985055

[84] Geter A Sutton MY Hubbard McCree D. Social and structural determinants of HIV treatment and care among black women living with HIV infection: a systematic review: 2005-2016. AIDS Care. (2018) 30:409–16. doi: 10.1080/09540121.2018.1426827, PMID: 29376409 PMC6459180

[85] Geter A Sutton MY Armon C Durham MD Palella FJ Jr Tedaldi E. Trends of racial and ethnic disparities in virologic suppression among women in the HIV outpatient study, USA, 2010-2015. PLoS One. (2018) 13:e0189973. doi: 10.1371/journal.pone.0189973, PMID: 29293632 PMC5749722

[86] Gelaude DJ Hart J Carey JW Denson D Erickson C Klein C. HIV provider experiences engaging and retaining patients in HIV care and treatment: "a soft place to fall". J Association Nurses AIDS Care: JANAC. (2017) 28:491–503. doi: 10.1016/j.jana.2017.03.006, PMID: 28442187 PMC5849236

[87] Byrd KK Hardnett F Clay PG Delpino A Hazen R Shankle MD. Retention in HIV care among participants in the patient-centered HIV care model: a collaboration between community-based pharmacists and primary medical providers. AIDS Patient Care STDs. (2019) 33:58–66. doi: 10.1089/apc.2018.0216, PMID: 30648888 PMC6379900

[88] Mimiaga MJ August Oddleifson D Meersman SC Silvia A Hughto JMW Landers S. Multilevel barriers to engagement in the HIV care continuum among residents of the state of Rhode Island living with HIV. AIDS Behav. (2020) 24:1133–50. doi: 10.1007/s10461-019-02677-4, PMID: 31563986 PMC7085442

[89] Wessinger MH Hennink MM Kaiser BN Mangal JP Gokhale RH Ruchin L. Retention in HIV care depends on patients' perceptions of the clinic experience. AIDS Care. (2017) 29:1212–7. doi: 10.1080/09540121.2017.1308465, PMID: 28366008 PMC7375480

[90] Aschbrenner KA Oh AY Tabak RG Hannon PA Angier HE Moore WT. Integrating a focus on health equity in implementation science: case examples from the national cancer institute's implementation science in cancer control centers (ISC(3)) network. J Clin Transl Sci. (2023) 7:e226. doi: 10.1017/cts.2023.638, PMID: 38028358 PMC10643915

[91] Shelton RC Adsul P Oh A Moise N Griffith DM. Application of an antiracism lens in the field of implementation science (IS): recommendations for reframing implementation research with a focus on justice and racial equity. Implement Res Pract. (2021) 2:26334895211049482. doi: 10.1177/26334895211049482, PMID: 37089985 PMC9978668

[92] Powell BJ Beidas RS Lewis CC Aarons GA McMillen JC Proctor EK. Methods to improve the selection and tailoring of implementation strategies. J Behav Health Serv Res. (2017) 44:177–94. doi: 10.1007/s11414-015-9475-6, PMID: 26289563 PMC4761530

[93] Powell BJ Waltz TJ Chinman MJ Damschroder LJ Smith JL Matthieu MM. A refined compilation of implementation strategies: results from the expert recommendations for implementing change (ERIC) project. Implement Sci. (2015) 10:21. doi: 10.1186/s13012-015-0209-1, PMID: 25889199 PMC4328074

[94] Garba RM Gadanya MA. The role of intervention mapping in designing disease prevention interventions: a systematic review of the literature. PLoS One. (2017) 12:e0174438. doi: 10.1371/journal.pone.0174438, PMID: 28358821 PMC5373531

[95] Durks D Fernandez-Llimos F Hossain LN Franco-Trigo L Benrimoj SI Sabater-Hernandez D. Use of intervention mapping to enhance health care professional practice: a systematic review. Health Educ Behav. (2017) 44:524–35. doi: 10.1177/1090198117709885, PMID: 28580805

