# Supplementary material for: St. Louis enhancing engagement and retention (STEER) in HIV/AIDS care: a participatory intersectional needs assessment for intervention and implementation planning
Source: Front Public Health. 2025 Jun 11;13:1589671. doi: 10.3389/fpubh.2025.1589671 (PMC12187841; doi:10.3389/fpubh.2025.1589671)
Supplement: Supplementary file 1 [file Supplementary_file_1.zip › Appendix A.docx]

**Clinical Leaders RAP Sheet**

Moderator:

Interviewee Number (e.g. CLIN1A):

Interview Date and Time:

Note Taker:

1. Peer Navigator Experiences

2. Strengths / Suggestions of Using Community Health Workers in St. Louis

3. Integration of CHWs into the Care Team

4. Challenges of Using CHWs in St. Louis

4. Barriers and Facilitators for retention in HIV Care

6. Other

**Community Leaders RAP Sheet**

Moderator:

Interviewee Number (e.g. COMM1A, CLIN1A):

Interview Date and Time:

Note Taker:

1A. Peer Navigator Experiences

2. Strengths / Suggestions of Using Community Health Workers in St. Louis

3. Challenges of Using CHWs in St. Louis

4. Barriers and Facilitators to HIV Care

5. Other

**People with Lived Experience RAP Sheet**

Moderator:

Interviewee Number:

Interview Date and Time:

1. Experiences with personal health
2. Experiences with health care system
3. Barriers for managing HIV care
4. Facilitators for managing HIV care
5. Strengths/Suggestions of Using Community Health Workers/front line health workers in St. Louis
6. Challenges of Using CHWs/front line workers in St. Louis
7. Other

**Focus Group RAP Sheet**

Moderator:

Focus Group Number:

Interview Date and Time:

Note Taker:

1. Front-line health workers titles, roles and responsibilities
2. Barriers affecting PLWHA’s access to HIV care
3. Challenges for front-line health workers
4. Front-line health workers’ support and relationship with other staff in the organization
5. Experience/thoughts on working with multi-organizational initiatives

**HRSA/BU CHWs & Supervisors RAP Sheet**

Interviewer:

Interviewee Number:

Interview Date and Time:

Primary Moderator / Secondary Moderator:

1A. CHW Experiences and Background

1B. Challenges and Impacts in HIV Care Management

2A. HRSA Strengths

2B. HRSA Challenges

2C. Supervision and Interaction with Organization

3A. Ongoing Client Barriers to HIV Care Management

3B. Adaptations Suggested to HRSA Intervention

4. Other
