# Supplementary material for: St. Louis enhancing engagement and retention (STEER) in HIV/AIDS care: a participatory intersectional needs assessment for intervention and implementation planning
Source: Front Public Health. 2025 Jun 11;13:1589671. doi: 10.3389/fpubh.2025.1589671 (PMC12187841; doi:10.3389/fpubh.2025.1589671)
Supplement: Supplementary file 1 [file Supplementary_file_1.zip › Appendix C.docx]

Appendix C: Explanations and support for pathways in linear model of the problem drawn from literature and interviews/focus groups.

1. Lack of sustainable funding often results in constraints on medical resources for providers to utilize in their patient interactions. Often the quality of patient care is compromised due to financial constraints.^1^
2. Institutions experiencing high provider turnover often end up with high caseloads for remaining providers. This results in more strain on providers^2^ and can lead to lower quality care for clients.
3. Lack of sustainable funding results in impaired financial capabilities of clinics to support providers, which can lead to professionals leaving their jobs.
4. The lack of robust training programs often leads to insufficient skill development in community health workers (CHWs), particularly around self-confidence in client interactions.^3^
5. Lack of robust training programs can also lead to a lack of HIV knowledge and practical skill set that is needed for client interactions. Some of these areas include biological mechanisms of HIV/AIDS, reading and interpreting patient charts, data recording, interviewing techniques, knowledge of available treatment options, and new research on HIV.^3^
6. Without robust training programs, providers may not receive sufficient preparation for handling complex situations with patients.
7. Insufficient pay and benefits can often put stress on providers to work effectively and can lead to unfavorable outcomes for patients.

8 -10. Lack of funding restricts the institutional capabilities to provide proper benefits and pay for their staff, the establishment of training programs for staff, and treatment plans for patients.

11. Hierarchical systems of communication lead to less integration of CHWs into care models.^4^ CHWs can feel excluded and unappreciated for their work.

12. The lack of integration of CHWs into the healthcare team leads to vague, poorly defined roles and responsibilities for CHWs. In addition, an undefined CHW role can also limit acceptability by other healthcare professionals.^4^ CHWs often feel they are not achieving their fullest potential in serving their clients.

13. CHWs' interactions and experiences with PLWH help inform, shape, and innovate treatment plans for HIV care. Insufficient training can limit the opportunities for CHWs to gather information and form a plan best suited to the client’s needs.

14. Providers’ input is vital to building training programs that help address deficiencies in the workforce skillset.

15. Lack of stable funding and sustainable forms of local incentives for CHWs limit their continuous involvement in the community. The long-term investment of CHWs to integrate themselves into the communities help build trust and better serve their needs.^5^

16. & 35. Insufficient time and attention to patients’ needs can prevent critical information from being gathered and implemented in making treatment plans. Limitations in treatment plans can hamper holistic care including inadequate attention to comorbidities.

17. & 18. Providers' personal beliefs and attitudes drive actions that leave PLWH feeling stigmatized. Often HIV clients felt judged, labeled, and stereotyped for their HIV status, which led to poorer relationships with providers such as treatment refusal and diagnosis denial.^6^

19. & 21. CHWs are often excluded from attaining knowledge and skills compared to other medical professions. This leads to restrictions in their roles and responsibilities such as home care visits to help address communities’ social needs.^7^

20. The lack of integration of CHW in the care model hinders the proper distribution of cases among the healthcare team, especially through a lack of case information sharing.

22 & 32. The inattention of patients’ needs from providers leads to more work needed from CHWs to address those needs. CHWs often feel overwhelmed by managing high caseloads which makes it difficult to evaluate and support clients in their care plan.^8^

23, 31, & 33. The limited scope of CHWs’ roles leads to a lack of confidence in what they wish to achieve and serve in their communities. Without the support and training, CHWs cannot gain the confidence to effectively address the social needs and barriers of their communities.

24. Clarification of CHWs' role and support from the institutions such as from CHW supervisors can help manage workloads properly and relieve workload burden for providers and CHWs.^7^

25, 28 & 29. Lack of confidence from CHWs can lead to insufficient social engagement with clients and understanding of their needs. When social needs are not addressed properly, it can result in outcomes like diagnosis denial and stigma.

26 & 27. Lack of knowledge of treatment paths hinders CHWs to navigate suitable treatment options for clients. Also, inadequate client engagement leads to imbalanced decision-making for clients when choosing the right treatment plan.

30. CHWs are seen as the bridge between the community and the healthcare system. The lack of integration of CHWs in community spaces results in a lack of trust and an ineffective ability to provide social support.

34. Inattention to patients’ needs from the provider can lead to lack of support to comorbidities such as diabetes and cardiovascular disease with clients living with HIV/AIDS.

References

1. Akinleye, D. D., McNutt, L.-A., Lazariu, V., & McLaughlin, C. C. (2019). Correlation between hospital finances and quality and safety of patient care. *PLoS ONE*, *14*(8), e0219124. <https://doi.org/10.1371/journal.pone.0219124>

2. Ginossar, T., Oetzel, J., Hill, R., Avila, M., Archiopoli, A., & Wilcox, B. (2014). HIV health-care providers’ burnout: Can organizational culture make a difference? *AIDS Care*, *26*(12), 1605–1608. <https://doi.org/10.1080/09540121.2014.936819>

3. Rajabiun, S., Baughman, A., Sullivan, M., Poteet, B., Downes, A., Davich, J. A. W., Phillips, S., Jackson, P., Miles, L., Drainoni, M.-L., Evans, E. M., Bachman, S. S., & Sprague Martinez, L. (2021). A Participatory Curricula for Community Health Workers and Supervisors to Increase HIV Health Outcomes. *Frontiers in Public Health*, *9*. <https://www.frontiersin.org/articles/10.3389/fpubh.2021.689798>

4. De Neve, J.-W., Boudreaux, C., Gill, R., Geldsetzer, P., Vaikath, M., Bärnighausen, T., & Bossert, T. J. (2017). Harmonizing community-based health worker programs for HIV: A narrative review and analytic framework. *Human Resources for Health*, *15*(1), 45. <https://doi.org/10.1186/s12960-017-0219-y>

5. Colvin, C. J., Hodgins, S., & Perry, H. B. (2021). Community health workers at the dawn of a new era: 8. Incentives and remuneration. *Health Research Policy and Systems*, *19*(3), 106. <https://doi.org/10.1186/s12961-021-00750-w>

6. Naughton, J. D., & Vanable, P. A. (2013). HIV Stigmatization Among Healthcare Providers: Review of the Evidence and Implications for HIV Care. In P. Liamputtong (Ed.), *Stigma, Discrimination and Living with HIV/AIDS: A Cross-Cultural Perspective* (pp. 97–114). Springer Netherlands. <https://doi.org/10.1007/978-94-007-6324-1_6>

7. Payne, J., Razi, S., Emery, K., Quattrone, W., & Tardif-Douglin, M. (2017). Integrating Community Health Workers (CHWs) into Health Care Organizations. *Journal of Community Health*, *42*(5), 983–990. <https://doi.org/10.1007/s10900-017-0345-4>

8. Lloyd, J., & Thomas-Henkel, C. (n.d.). *Integrating Community Health Workers into Complex Care Teams: Key Considerations*.
